# A HER2 Specific Nanobody–Drug Conjugate: Site-Selective Bioconjugation and In Vitro Evaluation in Breast Cancer Models

**DOI:** 10.3390/molecules30020391

**Published:** 2025-01-18

**Authors:** Anders H. Hansen, Kasper I. H. Andersen, Li Xin, Oliver Krigslund, Niels Behrendt, Lars H. Engelholm, Claus H. Bang-Bertelsen, Sanne Schoffelen, Katrine Qvortrup

**Affiliations:** 1 Department of Chemistry, Technical University of Denmark, 206 Kemitorvet, 2800 Kgs Lyngby, Denmark; anders.hoejgaard.hansen@gmail.com; 2 National Food Institute, Technical University of Denmark, 2800 Kgs Lyngby, Denmarkclaban@food.dtu.dk (C.H.B.-B.); 3 Department of Health Technology, Technical University of Denmark, 2800 Kgs Lyngby, Denmark; 4 Finsen Laboratory, Rigshospitalet/Biotech Research and Innovation Center (BRIC), University of Copenhagen, 2200 Copenhagen, Denmarkniels.behrendt@finsenlab.dk (N.B.); lhe@finsenlab.dk (L.H.E.); 5 The Novo Nordisk Foundation Center for Biosustainability, Technical University of Denmark, 2800 Kgs Lyngby, Denmark

**Keywords:** breast cancer, targeted treatment, nanobody, site-selective conjugation

## Abstract

A human epidermal growth factor receptor 2 (HER2)-specific nanobody called 2Rs15d, containing a His3LysHis6 segment at the C-terminus, was recombinantly produced. Subsequent site-selective acylation on the C-terminally activated lysine residue allowed installation of the cytotoxin monomethyl auristatin E-functionalized cathepsin B-sensitive payload to provide a highly homogenous nanobody–drug conjugate (NBC), which demonstrated high potency and selectivity for HER2-positive breast cancer models.

## 1. Introduction

Antibody-drug conjugates (ADCs) have shown great promise for treatment of antigen overexpressing cancers, as testified by the rapidly increasing number of approved ADCs in recent years [1]. Amidst the growing enthusiasm for ADCs, their design entails several challenges. One limitation is the large size of the ADC (~150 kDa) [2,3], which hampers penetration into deeper tissue, leading to large untargeted regions that escape therapy. Only few ADCs have entered Phase II clinical trials in solid tumor indications, suggesting that smaller delivery formats are warranted for such indications [4,5]. Furthermore, the Fc region of immunoglobulins (IgG) is capable of binding to both neonatal Fc-receptors and Fc-gamma receptors, events that are associated with undesired off-target toxicity [6]. Another limitation is the suboptimal conjugation technology [7]. Limited site-specificity leads to ADCs that are heterogeneous mixtures of chemically distinct molecules that vary in both the number and position of the cytotoxic agent. ADCs with suboptimal drug-to-antibody ratios are prone to aggregation, poor solubility, and instability, which often lead to increased toxicity or inadequate efficacy in vivo [8].

To date, smaller antibody variants such as single-chain variable fragments (scFv) and single-domain antibodies (sdAb) have, in their unconjugated form, been explored as an immunotherapeutic approach to cancer treatment [9]. Heavy-chain antibodies in camelids containing merely a single variable domain represent a group of small (~15 kDa) and yet highly antigen-specific proteins, also called nanobodies (Nbs) [10]. Upon binding to a target antigen, Nbs can act either as antagonists or as internalizing ligands [10]. Relative to full-sized antibodies and their drug conjugates [11], Nbs exhibit higher tissue penetration and faster clearance [12]. Owing to these intrinsic properties, Nb conjugates have traditionally been used for molecular imaging where quick elimination is desired. This application has previously been explored in a variety of cancers, e.g., in human epidermal growth factor receptor 2 (HER2)-positive cancers, where Nbs were employed as vectors for targeted α-particle therapy using both ^225^Ac and ^211^At as radionuclides [13,14]. Additionally, Nbs can be produced in non-mammalian host systems such as *E. coli*, contrasting the significantly higher costs associated with the manufacturing of full-sized monoclonal antibodies that necessitate mammalian cultures.

One approach to overcome the intrinsic properties of IgG formats while also increasing the clinical efficacy in solid tumors is to introduce Nbs and other small antibody formats with greater penetrative potential as carriers of highly cytotoxic compounds [15,16]. However, the reported use of Nbs as a generic carrier system for delivering cytotoxic payloads, analogously to that of the ADC platform, remains scarce. Previous examples include α3β1-specific scFv-duocarmycin conjugates [17], site-specifically modified Fab-MMAE conjugate for HER2-positive cancer [18], and a CA9-specific scFv-auristatin F conjugate [19]. In a more recent study, Aubrey et al. demonstrates the use of a second generation maleimide for site-specific conjugation of monomethyl auristatin F (MMAF) to an engineered HER2-specific scFv [20].

Herein, we report on an improved bioconjugation procedure for a previously reported HER2-specific Nb called 2Rs15d [21]. Specifically, the incorporation of a His3Lys His6 (H_3_KH_6_) handle in the C-terminus-enabled site-selective acylation on the C-terminally activated lysine residue. Subsequent attachment of the cytotoxin MMAE in two steps provided a highly homogenous, potent, and selective nanobody–drug conjugate (NBC) when evaluated in HER2-positive breast cancer models.

## 2. Results and Discussion

As a starting point, we were intrigued by the data reported by Massa et al. [21] demonstrating retained ligand function in a 2Rs15d variant containing a 14 amino acid-long spacer sequence (SPSTPPTPSPSTPPC) inserted into the C-terminal end of the nanobody (2Rs15d-HLC). Further, this spacer is terminated by a cysteine, thereby enabling conjugation with maleimide-functionalized compounds, such as the ligand pentetic acid [21]. Later, a more comprehensive work was published by the same group also disclosing structural information on the ligand receptor complexes [22]. Here, it was found that while pertuzumab and trastuzumab bind to domain II and IV on the HER2 receptor, respectively, 2Rs15d binds to domain I, and this observation aligned with the lack of competition found in vivo between 2Rs15d, pertuzumab, and trastuzumab.

**Figure 1:**
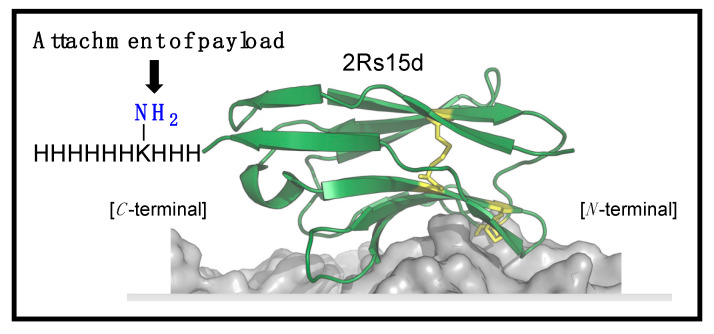
Structure of 2Rs15d tagged with H3KH6 at the C-terminus for site-selective conjugation.

Inspired by this, we aimed to functionalize 2Rs15d at the C-terminal. Identifying the potential challenges when working with proteins containing a free thiol, including the risk of protein dimerization [21,23], and/or disulfide bond scrambling [24], as well as the limited stability of maleimide–cysteine thioether linkages not being ideal for drug targeting conjugation [25], we opted to functionalize 2Rs15d with a lysine residue sandwiched in between His3 and His6 motifs, and primed for acylation (Figure 1) [26]. Importantly, this handle has a dual function, as it serves both as a His-tag for purification after recombinant synthesis, while also serving as a director for site-selective acylation and installation of an azido bioconjugation handle as reported previously [26]. As internal controls, we also produced 2Rs15d and 2Rs15d-HLC [21,22].

The production of 2Rs15d-H3KH6 was performed using a similar procedure as that for 2Rs15d and 2Rs15d-HLC in *E. coli* (see below and Supplementary Materials). Purification by immobilized metal-affinity chromatography followed by a size-exclusion chromatography step gave pure protein in a yield of 1–1.5 mg/L bacterial culture, as confirmed by SDS-PAGE analysis (Figures S1–S5).

**Figure 2:**
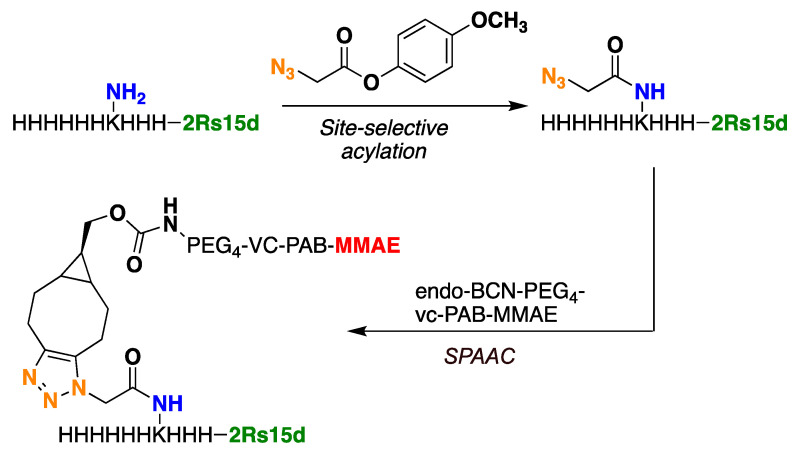
Bioconjugation of 2Rs15d-H_3_KH_6_ to BCN-based MMAE payload takes place in two steps; acylation and strain-promoted click reaction (SPAAC).

With the two Nbs, 2Rs15d-HLC and 2Rs15d-H_3_KH_6_, in hand and set up for maleimide-cysteine-based bioconjugation and Lys-His tag acylation followed by strain-promoted azide–alkyne cycloaddition (SPAAC) bioconjugation (Figure 2 and Figure S9), respectively, we set out to screen for optimal conditions to facilitate clean conversion to the desired monomethyl auristatin E (MMAE)-based conjugates 2Rs15d-HLC-MMAE and 2Rs15d-H_3_KH_6_-MMAE. While Massa and coworkers described maleimide–cysteine conjugation between 2Rs15d-HLC and a maleimide-functionalized dye, we were never able to observe successful thiosuccinimide formation when reacting 2Rs15d-HLC with the maleimide-functionalized payload mc-vc-PAB-MMAE. Although several conditions were utilized in testing (see Supplementary Materials, Table S1 and Figures S6–S8), we only observed nanobody dimerization, which was also observed as a byproduct by Massa and coworkers [21].

The 2Rs15d-H_3_KH_6_ protein, on the other hand, was readily acylated using 4-methoxy phenyl azido acetate. Various amounts of acylating reagents were tested. The degree of functionalization was assessed by intact-protein mass spectrometry. Typically, a mixture of starting materials and mono-acylated and di-acylated products were obtained (see Supplementary Materials, Figure S10). The more reagent was added, the lower the amounts of starting material and the larger the amounts of the di-acylated product relative to the mono-acylated product (see Supplementary Materials, Figure S11). It was decided that 40 equivalents of the reagent should be used for subsequent preparations of the acylated 2Rs15d-H_3_KH_6_ nanobody (40 μM protein vs. 1.6 mM reagent).

The acylated protein was digested with chymotrypsin and analyzed by mass spectrometry. This was done to confirm that the site-selectivity resides in the lysine residue within the H_3_KH_6_ motif (see Supplementary Materials, Table S2). While the chymotryptic peptide covering the H_3_KH_6_ tag was identified in acylated form only, two chymotryptic peptides covering other Lys residues of 2Rs15d were identified in a non-modified form, and also, to a lesser extent, in acylated form. The Lys residues responsible for off-site acylation are residues 62 and/or 66 and residues 77 and/or 78. No acylation was observed for the N-terminal amine, Lys residue 21, or Lys residue 88.

Next, azidated 2Rs15d-H_3_KH_6_ was subjected to SPAAC using the alkyne functionalized payload endo-BCN-PEG4-vc-PAB-MMAE (Figure 2 and Figure S9) [27]. The PEG4 unit was included to circumvent potential aggregation during NBC synthesis and to secure easy post-production handling of the final NBC. Furthermore, in a later study by Li et al., they demonstrate that incorporation of PEG units into linkers connecting a nanobody to MMAE accommodated a positive effect on the half-life of the conjugate and cytotoxicity, relative to a conjugate that lacks the PEG units [28]. The SPAAC reactions were successfully carried out using 20 mM Tris at pH 7.5 at room temperature. As a note, we observed precipitation of this payload at 5% DMSO content in 20 mM Tris, pH 7.5, where co-solvents were tested in addition to DMSO to remedy this problem. The best result was obtained when using a combination of dimethyl formamide DMF(5%) propylene glycol (PG, 20%), and DMSO (5%), which led to complete dissolution of all components and to the successful production of the conjugate 2Rs15d-H_3_KH_6_-MMAE (see Supplementary Materials, Figure S12). The conjugate containing a single payload molecule was separated from the smaller amounts of di-functionalized and unconjugated material by size-exclusion chromatography (see Supplementary Materials, Figures S13 and S14).

Next, we set out to study the HER2-dependent cytotoxicity of 2Rs15d-H_3_KH_6_-MMAE in vitro. Since only a few reports on NBCs exist in the literature, we were intrigued to learn how efficiently the nanobody could internalize the relatively large MMAE-based payload [27] with roughly one-tenth of the molecular weight of that of 2Rs15d. In order to examine the cytotoxic potential of HER2-directed 2Rs15d-H_3_KH_6_-MMAE in vitro, we selected three well-characterized breast cancer models, namely the HER2-positive cell lines SKBR3 [28] and BT474 [29], and the HER2-negative cell line MCF-7 [30]. SKBR3 and BT474 both have high HER2 expressions and show efficient internalization upon receptor activation [29], while MCF7 is an often-used HER2-negative control cell line [22]. All three cell lines are sensitive to MMAE (IC50 = 0.23, 0.57 and 0.5 nM, respectively) and all express cathepsins needed for cleavage of the linker and release MMAE in the lysosome [27].

**Figure 3:**
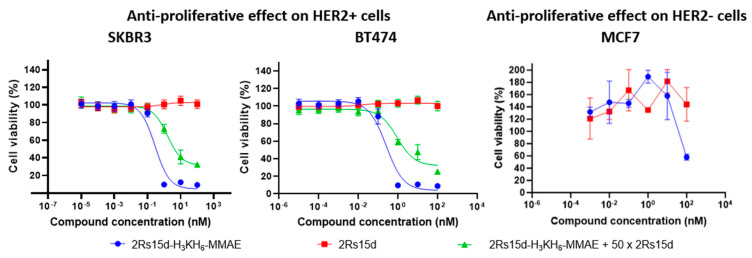
Anti-proliferative effect on HER2 + SKBR3 and BT474 cells as well as HER2-MCF7 cells.

Cells were treated with serially diluted 2Rs15d-H_3_KH_6_-MMAE, 2Rs15d (negative control), and the ADC trastuzumab-vc-MMAE (Tras-vc-MMAE, ~DAR 4, positive control), incubated for 4 days or 7 days, at which point cytotoxicity was evaluated by the MTS cell viability assay. Both HER2-positive cell lines displayed HER2-specific antibody-binding, conjugate internalization, and MMAE release from 2Rs15d-H_3_KH_6_-MMAE and Tras-vc-MMAE (Supplementary Materials, Figure S15), leading to efficient cell killing (for 2Rs15d-H_3_KH_6_-MMAE see Figure 3; for Tras-vc-MMAE, see Supplementary Materials, Figure S15). The ADC B12-vc-MMAE was used as a control conjugate to demonstrate the retained stability of the vc-linker during the incubation conditions employed for 2Rs15d-H_3_KH_6_-MMAE and Tras-vc-MMAE (Supplementary Materials, Figure S15). While the BT474 cell line showed a maximal loss in viability when evaluated on day 4, a 7-day incubation period was required for full effect in SKBR3 (Figure 3).

Notably, a smaller non-specific effect on the negative control cell line MCF-7 was observed only at the highest concentration (100 nM) of 2Rs15d-H_3_KH_6_-MMAE. However, the MCF-7 cell line otherwise retained cell viability in the concentration regime covered and no antiproliferative activity of 2Rs15d-H_3_KH_6_-MMAE was observed in MCF-7 as expected (Figure 3). HER2-mediated cytotoxicity was further demonstrated by coincubation with a 50-fold molar excess of unmodified Nb, which significantly inhibited binding and internalization of 2Rs15d-H_3_KH_6_-MMAE (Figure 3). Collectively, our data demonstrate high cytotoxicity and specificity of 2Rs15d-H_3_KH_6_-MMAE toward HER2-expressing cell lines. In addition, and since 2Rs15d binds to a different domain than trastuzumab, drug conjugates derived from 2Rs15d could potentially be employed in patients with resistance toward trastuzumab and T-DM1.

## 3. Materials and Methods

### 3.1. Nanobody Target Sequences

2Rs15d: MAQVQLQESGGGSVQAGGSLKLTCAASGYIFNSCGMGWYRQSPGRERELVSRISGDGDTWHKESVKGRFTISQDNVKKTLYLQMNSLKPEDTAVYFCAVCYNLETYWGQGTQVTVSSHHHHHH

2Rs15d-H_3_KH_6_: MAEVQLQESGGGSVQAGGSLKLTCAASGYIFNSCGMGWYRQSPGRERELVSRISGDGDTWHKESVKGRFTISQDNVKKTLYLQMNSLKPEDTAVYFCAVCYNLETYWGQGTQVTVSSHHHKHHHHHH

### 3.2. Plasmids for 2Rs15d and 2Rs15d-H_3_KH_6_

The plasmid for 2Rs15d was constructed by GenScript Biotech (Leiden, The Netherlands). The overall nanobody design was inspired by the original work reported by Massa et al. [21]. Briefly, the VHH sequence encoding the anti-HER2 nanobody 2Rs15d, codon-optimized for expression in *Escherichia coli* [28], was synthesized with a C-terminal His(6)-tag and flanking NcoI and XhoI restriction sites. Two other similar oligonucleotide sequences were constructed to also contain the linker sequence 5′-SPSTPPTPSPSTPP-3′, inserted either into the N-terminal or C-terminal of the nanobody sequences. Expression vector for periplasmic expression of His(6)-tagged nanobody was constructed by ligating the three oligonucleotide sequences into the respective pET-22b+ plasmids digested with NcoI and XhoI. The recombinant pET-22b+ plasmid with nanobody–His(6) fusions was transformed with heat shock into chemically competent *E. coli* One Shot^®^ BL21 (DE3) (Thermo Fisher, Waltham, MA, USA) for recombinant expressions and into chemically competent E. coli One Shot^®^ TOP10 (Thermo Fisher) for plasmid amplifications. Transformants were selected on Luria–Bertani (LB, Sigma, St. Louis, MO, USA) agar plates containing 100 µg/mL ampicillin.

Similarly to the plasmids encoding 2Rs15d and 2Rs15d-HLC, the gene fragment encoding 2Rs15d-H_3_KH_6_ was placed between the NcoI site and XhoI site in the pET-22b+ plasmid. The plasmid was transformed into chemically competent *E. coli* BL21 Star (DE3) (Thermo Fisher) for recombinant expression and *E. coli* Mach1 (Thermo Fisher) for plasmid amplification. Transformants were selected on Luria–Bertani (LB, Sigma) agar plates containing 100 µg/mL ampicillin.

### 3.3. Expression of 2Rs15d

Overnight cultures of transformants grown in LB medium (Sigma) containing 100 µg/mL ampicillin were diluted to an OD600 of 0.05–0.1 with 250 mL fresh Terrific Broth (TB) medium (Sigma) supplemented with 0.1% glucose (*w*/*v*), 1 mM MgCl_2_ and 100 µg/mL ampicillin and grown at 37 °C with agitation (180 rpm) until the culture reached an OD600 of 0.7. At this point nanobody expression was induced by adding 0.75 mM isopropyl β-d-1-thiogalactopyranoside (IPTG) and culture growth was continued at 28 °C for an additional 18 h with agitation (180 rpm). Subsequently, cells were harvested (9000× *g*, 10 min) at 4 °C and the pellet was kept frozen at −20 °C until its use.

### 3.4. Purification and Characterization of 2Rs15d

Frozen pellets were thawed on ice and then lysed by resuspending in 10 mL of lysis buffer (50 mM sodium phosphate, 300 mM sodium chloride, 10 mM imidazole; pH 7.4) containing 1 mg/mL lysozyme (Sigma) and 25 U/mL Benzonase^®^ Nuclease (Merck Millipore, Burlington, MA, USA) and incubated on ice for 30 min. Supernatant, containing soluble proteins, was separated from non-lysed bacteria and cell debris by high speed centrifugation (14,000× *g*, 30 min) at 4 °C. Supernatant was loaded onto a gravity-flow Fast Start Column (QIAGEN, Hilden, Germany) packed with 0.5 mL of Ni-NTA (nickel–nitrilotriacetic acid) resin and pre-equilibrated with 10 column volumes of buffer (50 mM sodium phosphate, 300 mM sodium chloride, 10 mM imidazole; pH 7.4). Each column was then washed twice with 4 mL of wash buffer (50 mM sodium phosphate, 300 mM sodium chloride, 50 mM imidazole; pH 7.4), followed by elution of the bound nanobody–His(6) proteins with elution buffer (50 mM sodium phosphate, 300 mM sodium chloride, 750 mM imidazole; pH 7.4) in two 1 mL fractions. Samples from individual washing and elution steps were analyzed on SDS-PAGE: 5 µL of sample was mixed with 10 µL ultra-pure water and 5 µL Laemmli Sample Buffer (Bio-Rad, Hercules, CA, USA) and 2-mercaptoethanol was added to a final concentration of 355 mM. The samples were boiled for 10 min and then loaded onto 16.5% precast polyacrylamide gels (Bio-Rad) and run using a Mini-PROTEAN Tetra Cell electrophoresis system (Bio-Rad). Protein bands were visualized on gels by incubation overnight in InstantBlue^TM^ Protein Stain (Expedeon, Cambridge, UK). Eluted fractions had their buffers exchanged to HEPES buffer (20 mM HEPES, 115 mM NaCl, 1.2 mM CaCl_2_, 1.2 mM MgCl_2_, 2.4 mM K_2_HPO_4_, pH 7.4) and were pooled and concentrated using 3 kDa centrifugal filter units (Amicon Ultra 0.5 mL, Merck Millipore). Subsequently, concentrated nanobody–His(6) fractions were loaded at a flow rate of 0.8 mL/min onto a Gel-filtration column (Superdex 75 10/300 GL, GE Healthcare Life Sciences, Chicago, IL, USA) pre-equilibrated with 48 mL of 20 mM HEPES buffer, pH 7.4. Fractions of 0.5 mL, corresponding to observed peaks at 280 nm and 214 nm, were collected and analyzed for size and purity on SDS-PAGE (see Supplementary Materials, Figure S1). Pure fractions corresponding to the size of monomeric nanobody–His(6) were pooled and concentrated using 3 kDa centrifugal filter units (Amicon Ultra-0.5 mL, Merck Millipore), and final protein concentration was determined using a Nanodrop spectrophotometer (Thermo Fisher Scientific). Protein fractions were kept at −80 °C until further use.

### 3.5. Mass Spectrometry Analysis of 2Rs15d

Intact protein mass spectrometry was performed on a Dionex UltiMate 3000 (Thermo Scientific) equipped with an Acclaim™ RSLC 120 C18 column (2.2 μm, 120 Å, 2.1 × 100 mm) coupled to a Bruker micrOTOF-QIII mass spectrometer (Billerica, MA, USA). A linear gradient of CH_3_CN in H_2_O with 0.1% formic acid was used, running from 5% to 100% CH_3_CN, at 0.5 mL/min over 10 min. Reactions were analyzed by electrospray ionization mass spectrometry and data were processed in Bruker Compass DataAnalysis. 2Rs15d; Calculated isotopic average: MW = 13,651.1 Da; Observed: 13,651.0 Da (supporting information Figure S2).

### 3.6. Expression of 2Rs15d-H_3_KH_6_

Overnight cultures of transformants grown in 2xYT medium (Sigma) containing 100 µg/mL ampicillin were diluted to an OD600 of 0.1 with 1 L fresh 2xYT medium supplemented with 100 µg/mL ampicillin and grown at 37 °C with agitation (120 rpm) until the culture reached an OD600 of 0.8. At this point, nanobody expression was induced by adding 1 mM isopropyl β-d-1-thiogalactopyranoside (IPTG) and culture growth was continued at 37 °C for an additional 3.5 h with agitation (120 rpm). Subsequently, cells were harvested (4800× *g*, 15 min) at 4 °C and the pellet was kept frozen at −20 °C until use.

### 3.7. Purification of 2Rs15d-H_3_KH_6_

Frozen pellets were thawed on ice and then lysed by resuspending in 35 mL of lysis buffer (20 mM sodium phosphate, 500 mM sodium chloride, 20 mM imidazole; pH 7.4) containing 1 mg/mL lysozyme (Sigma), 50 U/mL Benzonase^®^ Nuclease (Sigma) and cOmplete™ EDTA-free protease inhibitor cocktail (Roche, Basel, Switzerland). The lysate was incubated on ice for 60 min and passed three times through an Avestin EmulsiFlex homogenizer (Ottawa, ON, Canada). Supernatant, containing soluble proteins, was separated from non-lysed bacteria and cell debris by high-speed centrifugation (15,000× *g*, 20 min) at 4 °C. Supernatant was loaded onto a 1 mL HisTrap FF column (Cytiva, Marlborough, MA, USA), which was pre-equilibrated with lysis buffer (20 mM sodium phosphate, 500 mM sodium chloride, 20 mM imidazole; pH 7.4). The column was then washed with 10 mL of lysis buffer and 15 mL of wash buffer (as lysis buffer, but with 35 mM imidazole). The bound protein was eluted with 10 mL of elution buffer (20 mM sodium phosphate, 500 mM sodium chloride, 1 M imidazole; pH 7.4). 0.5 mL fractions were collected. Elution fractions containing the protein of interest (as confirmed by SDS-PAGE analysis) were pooled and concentrated using 3 kDa centrifugal filter units (Amicon Ultra-4 mL, Merck Millipore). Subsequently, the concentrated protein solution was loaded onto a gel filtration column (Superdex 75 increase 10/300 GL, GE Healthcare) using PBS buffer (50 mM sodium phophaste, 150 mM NaCl, pH 7.5) as eluent. Pure fractions corresponding to the size of monomeric nanobody–His(6) were pooled and concentrated using a 3 kDa centrifugal filter unit (Amicon Ultra-4 mL, Merck Millipore) The final protein concentration was determined using a Nanodrop spectrophotometer (Thermo Fisher Scientific) and the purity and identity were assessed by SDS-PAGE analysis and intact-protein MS analysis, respectively (see Figure S5). Protein fractions were kept at −20 °C until further use.

### 3.8. Bioconjugation Protocol for 2Rs15d Containing H_3_KH_6_ Tag and BCN-Based MMAE Payload

Step 1—acylation procedure: The acylation between 2Rs15d-H_3_KH_6_ and 4-methoxyphenyl 2-azidoacetate was performed based on a procedure literature (step 1, Figure S9) [23] as follows: in an Eppendorf tube, 4-methoxyphenyl 2-azidoacetate (14 mM, 40 equiv) in acetonitrile was added to a solution of 2Rs15d-H_3_KH_6_ (0.5 mg/mL) in 20 mM Tris (pH 7.5) at 4 °C. The reaction was run for 1 day at 4 °C, after which excess 4-methoxyphenyl 2-azidoacetate was removed using 7 kDa MWCO Zeba Spin (Thermo Scientific) pre-equilibrated with 20 mM Tris (pH 7.5). The recovered azidated 2Rs15d-H_3_KH_6_ (0.4 mg/mL) was further concentrated (Amicon, MWCO 3 kDa) until a protein concentration of 1.75 mg/mL was obtained, and the sample was used as such without further purification in the next step.

Analysis of the intact protein by reverse-phase HPLC-MS demonstrated successful functionalization. The differently substituted products were not separatable under the LC conditions used (Poroshell 120 SB-C8 column (Agilent, Santa Clara, CA, USA), 2.7 µm, 2.1 × 50 mm, linear gradient of MeCN in water with 0.1% formic acid and 2.5 mM NH_4_OH, from 0 to 100% over 9 min). Typically, a mixture of mono-acylated and di-acylated products was obtained with minor amounts of starting material and triacylated product (Figures S10 and S11).

Step 2—strain-promoted click procedure: Based on initial screening (see Supplementary Materials, incl Figure S12), the click reaction between acylated 2Rs15d-H_3_KH_6_ and endo-BCN-PEG4-vc-PAB-MMAE (BroadPharm, San Diego, CA, USA) was performed in 20 mM Tris pH 7.5 at room temperature (step 2, Figure S9). The payload endo-BCN-PEG4-vc-PAB-MMAE (20 equivalents) in DMSO (2.4 mM) was added to acylated 2Rs15d-H_3_KH_6_. To avoid precipitation of payload during these conditions, the co-solvents dimethyl formamide (DMF, 5%) and propylene glycol (PG, 20%) were found to be optimal additives to ensure complete dissolution of the payload during the click reaction. Briefly, endo-BCN-PEG4-vc-PAB-MMAE (20 equivalents) was added to azidated 2Rs15d-H_3_KH_6_ (1.2 mg/mL) in 20 mM Tris (pH 7.5). Next, DMF (5% *v*/*v*) and PG (20% *v*/*v*) were added, and each reaction was adjusted to a final protein concentration of 0.7 mg/mL using 20 mM Tris (pH 7.5). The reaction mixture was degassed, and the reaction was shaken (500 rpm) on a thermomixer (37 °C) for 48 h. Next, excess endo-BCN-PEG4-vc-PAB-MMAE was removed using 7 KDa MWCO Zeba Spin pre-equilibrated with 20 mM Tris (pH 7.5). In order to separate unreacted azidated 2Rs15d-H_3_KH_6_ from the desired conjugate 2Rs15d- H_3_KH_6_-MMAE, the reaction Fractions mixture (devoid of free unreacted payload) was subjected to size-exclusion chromatography on a Superdex75 10/300 GL column (GE Healthcare) using PBS as eluent (Figure S13a). Fractions (0.2 mL each) were subjected to SDS-PAGE analysis followed by Western blotting using an anti-His antibody (Invitrogen) (Figure S13b). Fraction f21, containing the pure mono-functionalized conjugate, as confirmed by intact-protein MS analysis (Figure S14), was selected for cytotoxicity studies.

### 3.9. Chymotrypsin Digestion and LC-MS/MS Analysis of Acylated 2Rs15d-H_3_KH_6_

The crude reaction mixture containing 2Rs15d- H_3_KH_6_ and 4-methoxyphenyl 2-azidoacetate was digested with chymotrypsin. The digests, prepared in duplicate, were subjected to LC-MS/MS analysis. Peptides were loaded onto a 2cm C18 trap column (ThermoFisher 164946), connected in-line to a 15 cm C18 reverse-phase analytical column (Thermo EasySpray ES904) using 100% Buffer A (0.1% Formic acid in water) at 750 bar, using the Thermo EasyLC 1200 HPLC system, and a column oven operating at 35 °C. Peptides were eluted over a 70 min gradient ranging from 6 to 60% Buffer B (80% acetonitrile, 0.1% formic acid) at 250 nl/min, and the Q-Exactive instrument (Thermo Fisher Scientific) was run using the DD-MS2 top10 method. Full MS spectra were collected at a resolution of 70,000, with an AGC target of 3 × 10^6^ or maximum injection time of 20 ms and a scan range of 300–1750 *m*/*z*. The MS2 spectra were obtained at a resolution of 17,500, with an AGC target value of 1 × 10^6^ or maximum injection time of 60 ms, a normalized collision energy of 25 and an intensity threshold of 1.7e4. Dynamic exclusion was set to 60 s, and ions with a charge state of <2 or unknown were excluded. MS performance was verified for consistency by running complex cell lysate quality control standards, and chromatography was monitored to check for reproducibility.

All raw LC-MS/MS data files were processed together using Proteome Discoverer version 2.4 (Thermo). In the processing step, Oxidation (M), Acylation (K), and protein N-termini acylation and Met-loss were set as dynamic modifications. All results were filtered with 0.05 delta Cn for PSMs. SequestHT was used as a database, matching spectra against the E.coli database from Uniprot together with the recombinant sequence. The numbers of PSMs covering the N-terminus and Lys residues in 2Rs15d-H_3_KH_6_, unmodified or acylated, are listed in Table S2.

### 3.10. HER2 Positive and Control Cell Lines

Cell lines positive for the HER2 receptor SKBR3 (ATCC, Catalog No. HTB-30) and BT474 (ATCC, Catalog No. HTB-20) were maintained in McCoy’s 5A medium and in Dulbecco’s Modified Eagle Medium (DMEM), respectively. The HER2-negative cell line MCF-7 (ATCC, Catalog No. HTB-22) was maintained in Eagle’s Minimum Essential Medium (EMEM). All media were supplemented with 10% FBS and 1% penicillin/streptomycin. Cells were incubated at 37 °C/5% CO_2_ and harvested at 50–75% confluency.

### 3.11. Cytotoxicity of 2Rs15d-H_3_KH_6_-MMAE In Vitro Cell Viability Assay

The viability assay procedure was inspired largely from a procedure in the literature [31]. Briefly, in a 96-well plate (Costar, Washington, DC, USA), cells were seeded out (1 × 10^3^ cells/well) at a total volume of 90 uL culture medium. After 24 h, the old medium was removed, and fresh medium was added along with serially diluted 2Rs15d-H_3_KH_6_-MMAE and 2Rs15d-H_3_KH_6_ (unconjugated control) with a maximum final nanobody concentration of 10 nM (10% PBS *v*/*v*). Cells were incubated for either 4 days or 7 days, at which point overall viability was evaluated by adding 15 µL CellTiter 96 AQueous One Solution Cell Proliferation Assay (MTS) (Promega, Madison, WI, USA). The 96-well plate was incubated for an additional 60 min, and the plate was read at 490 nm (background subtraction at 630 nm), and viability was calculated as the percentage of internally untreated control cells.

## 4. Conclusions

In summary, we report on a human epidermal growth factor receptor 2 (HER2)-specific nanobody called 2Rs15d containing a His3LysHis6 segment at the C-terminus, allowing site-selective installation of the cytotoxin monomethyl auristatin E-functionalized cathepsin B-sensitive payload to provide a highly homogenous nanobody–drug conjugate, which demonstrated high potency and selectivity for HER2-positive breast cancer models.

We believe that the Lys-His tag acylation method for site-selective bioconjugation of nanobodies holds great promise—not only for the production of NBCs, but also for nanobodies used in imaging or as assay reagents, as well as for the oriented immobilization of nanobodies used to study protein–protein interactions or as biosensors.

The option to use lysine-directed acylation instead of a cysteine-maleimide coupling eliminates the need to work with a nanobody that is prone to dimerization, as we observed to be the case for 2Rs15d-HLC. Moreover, the C-terminal H_3_KH_6_ tag represents a tag with dual functionality. It directs the acylation to one specific Lys residue and at the same time functions as purification tag.

While site-selective bioconjugation to Lys residues in proteins are generally compromised by the larger number of native Lys residues generally present in proteins, the total number of Lys residues in nanobodies is small. In cases where off-site acylation occurs for a limited number of residues (as turned out to be the case for 2Rs15d), the process of protein engineering to remove these liabilities will be straightforward. The available knowledge of the nanobody structure (alone or in a complex with its target), sequence alignments and the possibility to predict changes in conformation when mutations are introduced will guide such an engineering process, enabling the fast development of a candidate that will be acylated at a single site only.

## Data Availability

All original data presented in the study are available upon request from the corresponding author.

## Supplementary Materials

The following supporting information can be downloaded at https://www.mdpi.com/article/10.3390/molecules30020391/s1: Experimental methods, nucleotide sequences, supplementary figures and tables (PDF).
