# Supplementary material for: A HER2 Specific Nanobody–Drug Conjugate: Site-Selective Bioconjugation and In Vitro Evaluation in Breast Cancer Models
Source: Molecules. 2025 Jan 18;30(2):391. doi: 10.3390/molecules30020391 (PMC11768052; doi:10.3390/molecules30020391)
Supplement: Supplementary file 1 [file molecules-30-00391-s001.zip › molecules-3343370-supplementary.pdf]

A HER2 Specific Nanobody-Drug Conjugate: Site-Selective Bioconjugation and *In Vitro* Evaluation in Breast Cancer Models

Anders H. Hansen[a], Kasper I. H. Andersen[b], Li Xin[c], Oliver Krigslund[d], Niels Behrendt[d], Lars H. Engelholm[d], Claus H. Bang-Bertelsen[b], Sanne Schoffelen[e], Katrine M. Qvortrup[a]

[a]*Department of Chemistry, Technical University of Denmark, 2800 Kgs Lyngby, Denmark* [b]*National Food Institute, Technical University of Denmark, 2800 Kgs Lyngby, Denmark* [c]*Department of Health Technology, Technical University of Denmark, 2800 Kgs Lyngby, Denmark.* [d]*Finsen Laboratory, Rigshospitalet/Biotech Research and Innovation Center (BRIC), University of Copenhagen, 2200 Copenhagen*

[e]*The Novo Nordisk Foundation Center for Biosustainability, Technical University of Denmark, Kongens Lyngby, Denmark*

2Rs15d-HLC nanobody target sequence **2**

Expression, purification and characterization of cysteine tagged variant 2Rs15d-HLC **2**

SDS-PAGE analysis and intact-protein MS analysis of 2Rs15d-H_3KH_6 .... **6**

Bioconjugation protocol for 2Rs15d-HLC and maleimide based MMAE payload **7**

Bioconjugation protocol for 2Rs15d containing H_3KH_6 tag and BCN-based MMAE payload **10**

Cytotoxicity of 2Rs15d-H_3KH_6-MMAE in vitro cell viability assay **15**

References **15**

2Rs15d-HLC nanobody target sequence

2Rs15d-HLC

MAEVQLQESG GGSVQAGGSL KLTCAASGYI FNSCGMGWYR QSPGRERELV SRISGDGDTW HKESVKGRFT ISQDNVKKTL YLQMNSLKPE DTAVYFCAVC YNLETYWGQG TQVTVSSHHH HHHSPSTPPT PSPSTPPC

Expression, purification and characterization of cysteine tagged variant 2Rs15d-HLC

Plasmids, oligonucleotides and bacterial strains

Plasmid for 2Rs15d-HLC were constructed by GenScript Biotech (Leiden, Netherlands). The overall nanobody design was inspired by the original work reported by Massa et al.[1] Briefly, the VHH sequence encoding the anti-HER2 nanobody 2Rs15d, codon-optimized for expression in *Escherichia coli* (D'Huyvetter et al.)h,[2] was synthesized with C-terminal $His_{(6)}$-tag and flanking *Nco*I and *Xho*I restriction sites. Two other similar oligonucleotide sequences were constructed to also contain the linker sequence 5'-SPSTPPTPSPSTPP-3' inserted either into the N-terminal or C-terminal of the nanobody sequences. Expression vectors for periplasmic expression of $His_{(6)}$-tagged nanobodies were constructed by ligating the three oligonucleotide sequences into their respective pET-22b+ plasmids digested with *Nco*I and *Xho*I. The recombinant pET-22b+ plasmids with nanobody-$His_{(6)}$ fusions were transformed with Heat Shock into chemically competent *E. coli* One Shot® BL21 (DE3) (ThermoFisher) for recombinant expressions and into chemically competent *E. coli* One Shot® TOP10 (ThermoFisher) for plasmid amplifications. Transformants were selected on Luria-Bertani (LB, Sigma) agar plates containing 100 µg/mL ampicillin.

Expression procedure

Overnight cultures of transformants grown in LB medium (Sigma) containing 100 µg/mL ampicillin were diluted to an OD_{600} of 0.05-0.1 with 250 mL fresh Terrific Broth (TB) medium (Sigma) supplemented with 0.1% glucose (w/v), 1 mM $MgCl_2$ and 100 µg/mL ampicillin and grown at 37 °C with agitation (180 rpm) until the culture reached an OD_{600} of 0.7. At this point nanobody expression

was induced by adding 0.75 mM isopropyl β-D-1-thiogalactopyranoside (IPTG) and culture growth was continued at 28 °C for an additional 18 hours with agitation (180 rpm). Subsequently, cells were harvested (9,000 *x g*, 10 minutes) at 4 °C and pellet was kept frozen at -20 °C until its use.

Purification procedure

Frozen pellets were thawed on ice and then lysed by resuspending in 10 mL of lysis buffer (50 mM sodium phosphate, 300 mM sodium chloride, 10 mM imidazole; pH 7.4) containing 1 mg/mL lysozyme (Sigma) and 25 U/mL Benzonase® Nuclease (Merck Millipore) and incubating on ice for 30 min. Supernatant, containing soluble proteins, was separated from non-lysed bacteria and cell debris by high speed centrifugation (14,000 *x g*, 30 minutes) at 4 °C. Supernatant was loaded onto a gravity-flow Fast Start Column (QIAGEN) packed with 0.5 mL of Ni-NTA (nickel-nitrilotriacetic acid) resin and pre-equilibrated with 10 column volumes of buffer (50 mM sodium phosphate, 300 mM sodium chloride, 10 mM imidazole; pH 7.4). Column was then washed twice with 4 mL of wash buffer (50 mM sodium phosphate, 300 mM sodium chloride, 50 mM imidazole; pH 7.4), followed by elution of the bound nanobody-$His_{(6)}$ proteins with elution buffer (50 mM sodium phosphate, 300 mM sodium chloride, 750 mM imidazole; pH 7.4) in two 1 mL fractions. Samples from individual washing and elution steps were analyzed on SDS-PAGE: 5 µL of sample was mixed with 10 µL ultra-pure water and 5 µL Laemmli Sample Buffer (Bio-Rad) and 2-mercaptoethanol was added to a final concentration of 355 mM. Samples were boiled for 10 minutes and then loaded onto 16.5% precast polyacrylamide gels (Bio-rad) and run using a Mini-PROTEAN Tetra Cell electrophoresis system (Bio-Rad). Protein bands were visualized on gels by incubating overnight in InstantBlue™ Protein Stain (Expedeon). Eluted fractions had their buffers exchanged to HEPES buffer (20 mM HEPES, 115 mM NaCl, 1.2 mM $CaCl_2$, 1.2 mM $MgCl_2$, 2.4 mM K_2HPO_4, pH 7.4) and were pooled and concentrated using 3-kDa centrifugal filter units (Amicon Ultra-0.5 mL, Merck Millipore). Subsequently, concentrated nanobody-$His_{(6)}$ fractions were loaded at a flow rate of 0.8 mL/min onto a Gel-filtration column (Superdex 75 10/300 GL, GE Healthcare Life Sciences) pre-equilibrated with 48 mL of 20 mM HEPES buffer, pH 7.4. Fractions of 0.5 mL, corresponding to observed peaks at 280 nm and 214 nm, were collected and analyzed for size and purity on SDS-PAGE. Pure fractions corresponding to the size of monomeric nanobody-$His_{(6)}$ were pooled and concentrated using 3-kDa centrifugal filter units (Amicon Ultra-0.5 mL, Merck Millipore) and final protein concentration was

determined using a Nanodrop spectrophotometer (Thermo Fisher Scientific). Protein fractions were kept at -80 °C until further use.

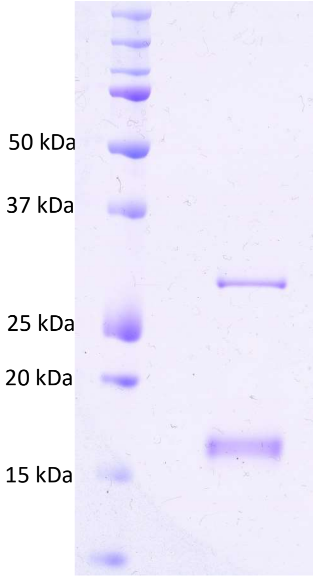

Figure S1: SDS page gel of purified 2Rs15d-HLC.

Mass Spectrometry of 2Rs15d and 2Rs15d-HLC

Intact protein analysis was performed on a Dionex UltiMate 3000 (Thermo Scientific) equipped with an Acclaim™ RSLC 120 C18 column (2.2 µm, 120Å, 2.1 x 100 mm) coupled to a Bruker micrOTOF-QIII mass spectrometer. A linear gradient of CH_3CN in H_2O with 0.1% formic acid was used, running from 5% to 100% CH_3CN, 0.5 mL/min over 10 min. Reactions were analyzed by electrospray ionization mass spectrometry and data processed in Bruker Compass DataAnalysis.

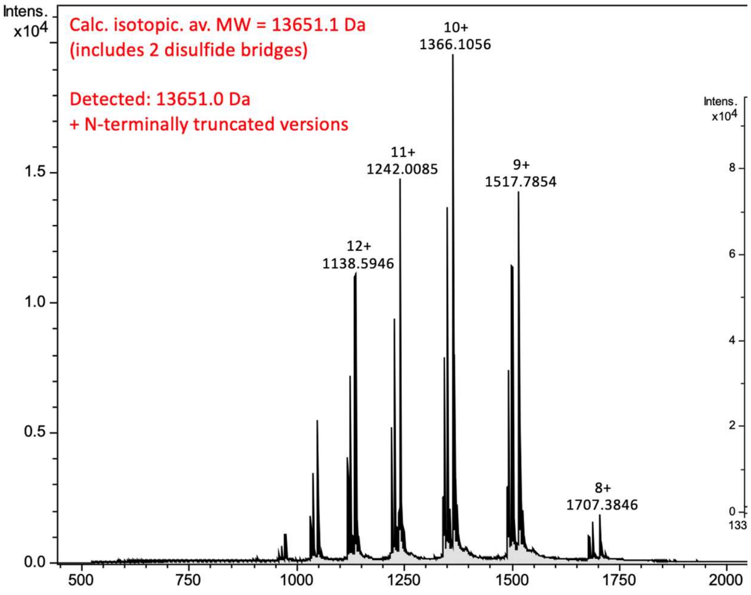

Figure S2: ESI-TOF spectrum of 2Rs15d.

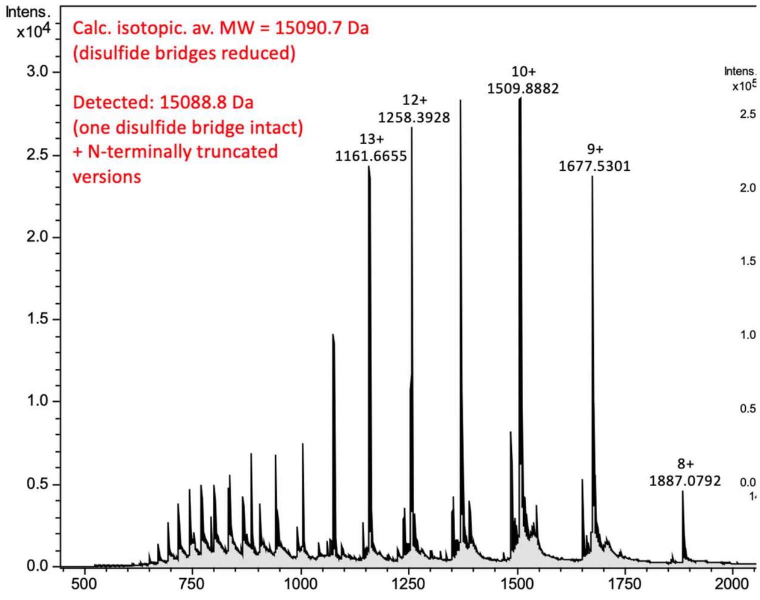

Figure S3: ESI-TOF spectrum of freshly reduced 2Rs15d-HLC using 5 mM DTT.

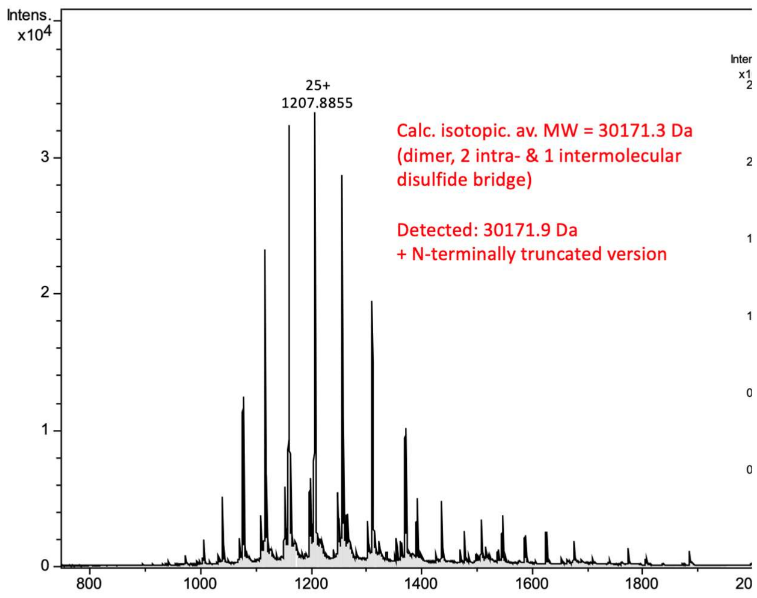

Figure S4: ESI-TOF spectrum of non-reduced dimerized 2Rs15d-HLC.

SDS-PAGE analysis and intact-protein MS analysis of 2Rs15d-H_3*K*H_6

SDS-PAGE analysis and intact-protein MS analysis of the final 2Rs15d-H_3*K*H_6 nanobody.

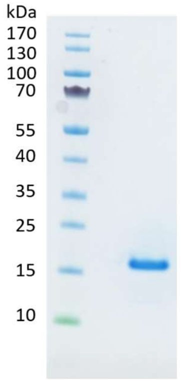

Figure S5a: SDS-PAGE gel of purified 2Rs15d-H_3*K*H_6.

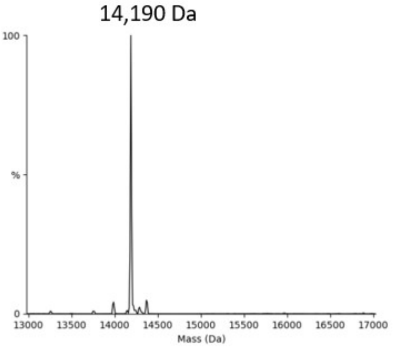

Figure S5b. Deconvoluted ESI-TOF spectrum of 2Rs15d-H_3*K*H_6.

Bioconjugation protocol for 2Rs15d-HLC and maleimide based MMAE payload

Overall, attempted bioconjugations between 2Rs15d-HLC and mc-vc-PAB-MMAE (Creative Biolabs) were performed based on a literature procedure.[1]

Briefly, to a solution of 2Rs15d-HLC in PBS (pH 7.4, 5 mM EDTA) was added a 180-fold 2-mercaptoethanolaminde (2-MEA). At a final protein concentration of 1.0 mg/mL, the reaction mixture was heated to 37 °C for 90 min. Next, using 7K ZebaSpin (Thermo Scientific), the freshly reduced protein was subjected to buffer exchange into 0.2 M NH_4OAc (pH 6.5, 5 mM EDTA). To the reduced 2Rs15d-HLC, 10 equivalents of mc-vc-PAB-MMAE in DMSO (7.6 mM) was added, gently mixed, and the bioconjugation between 2Rs15d-HLC and mc-vc-PAB-MMAE was allowed to proceed for 2 h at 37 °C. Finally, excess mc-vc-PAB-MMAE was removed (7K ZebaSpin) and the protein buffer-exchanged into PBS (pH 7.4) for further analysis using SDS PAGE.

Table S1: Screening conditions for bioconjugations using 2Rs15d-HLC and mc-vc-PAB-MMAE including conditions reported in literature (Entry 1).

Entry	Amount of mc-vc-PAB-MMAE (equiv)	Reducing agent	Temperature during bioconjugation (°C)
1[1]	10	2-MEA (180 equiv)	37
2	2	No reduction	rt
3	10	No reduction	rt
4	2	No reduction	37
5	10	No reduction	37
6[a]	10	TCEP (5 equiv)	37
7[a]	10	TCEP (5 equiv)	rt

TCEP: (Tris(2-carboxyethyl)phosphine).

[a]Treating the protein with TCEP for 30 min. TCEP was not removed prior to initiating the bioconjugation reaction.

The bioconjugation screening was analyzed using SDS PAGE (NUPAGE 12% Bis-Tris gel (Life technologies) in MOPS buffer.

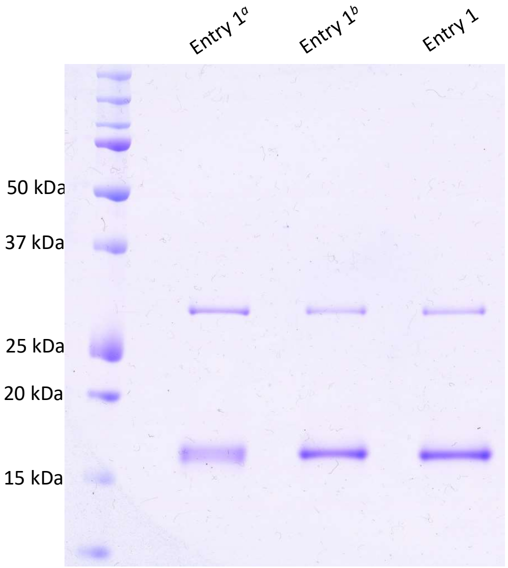

Figure S6: SDS page gel of nanobody prior to reducion (a), after reduction (b), and after bioconjugation (1) using the reported bioconjugation conditions.

Entry 2 Entry 3 Entry 4 Entry 5 Reduced 2Rs15d-HLC TBZ

50 kDa

37 kDa

25 kDa

20 kDa

15 kDa

50 kDa (heavy chain)

2Rs15d-HLC dimer

25 kDa (light chain)

2Rs15d-HLC (monomer)

Figure S7: SDS page gel of bioconjugation attempts using various conditions listed in Tabel S1, Entry 2-5. TZB: Trastuzumab (internal control).

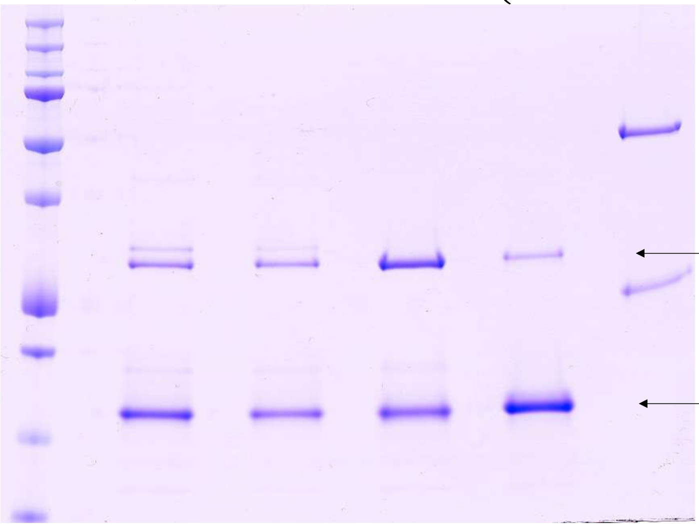

Figure S8: SDS page gel of bioconjugation attempts using various conditions listed in Tabel S1, Entry 6-7. TZB: Trastuzumab (internal controgl).

Bioconjugation protocol for 2Rs15d containing H_3KH_6 tag and BCN-based MMAE payload

Overview of the bioconjugation protocol for 2Rs15d-H_3KH_6 comprises two steps: (step 1) Acylation with 4-methoxyphenyl 2-azidoacetate (20 to 40 equivalents of acylating reagent)[3], (step 2) Click reaction between acylated 2Rs15d-H_3KH_6 and endo-BCN-PEG$_4$-vc-PAB-MMAE (BroadPharm), Figure S9.

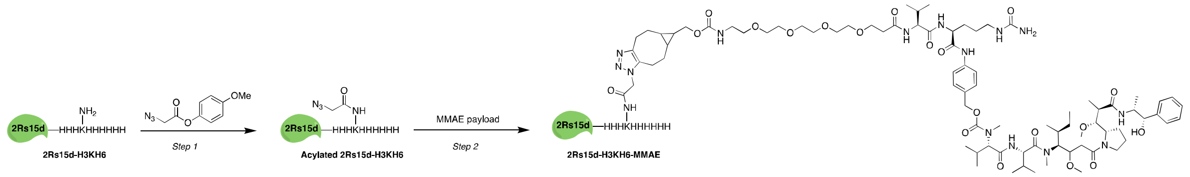

Figure S9: Bioconjugation of 2Rs15d-H_3KH_6 to BCN-based MMAE payload takes place in two steps; acylation (step 1) and strain promoted click reaction (step 2).

Analysis of the intact protein after the acylation (step 1) by reverse-phase HPLC-MS demonstrated successful functionalization. Typically, a mixture of mono-acylated and di-acylated product was obtained with minor amounts of starting material and triacylated product (Figure S10, S11).

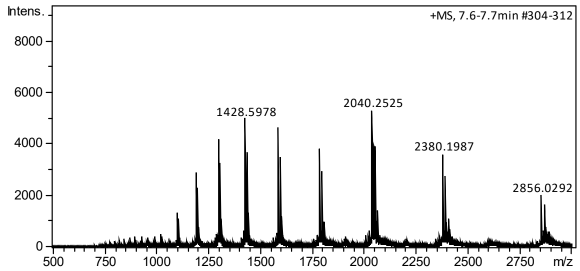

Figure S10a: ESI-TOF spectrum after acylation of 2Rs15d-H_3KH_6 with 40 eq. of acylating reagent.

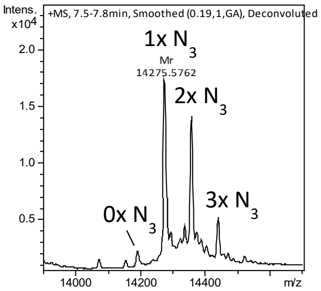

Figure S10b: Deconvoluted ESI-TOF spectrum after acylation of 2Rs15d-H_3KH_6 with 40 eq. of acylating reagent.

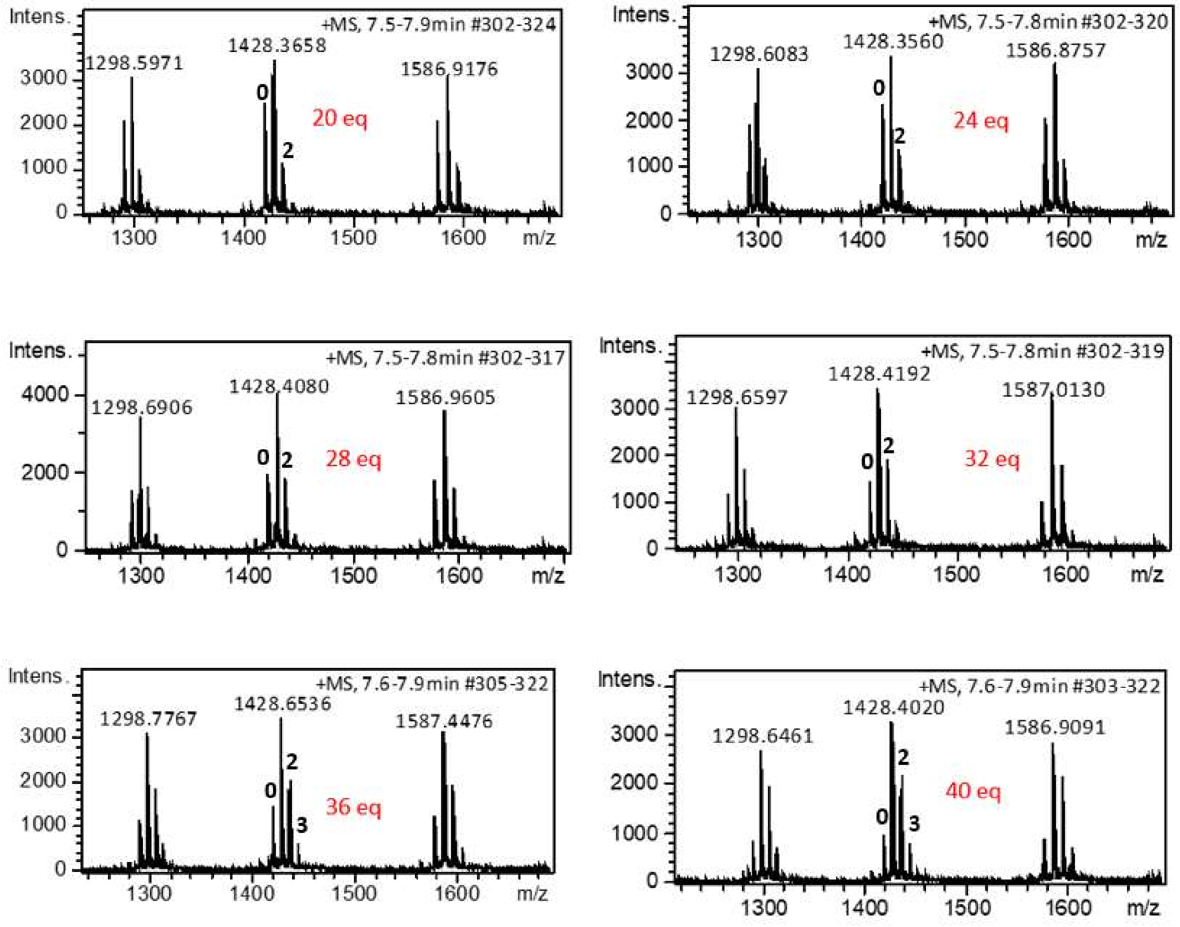

Figure S11: ESI-TOF spectra after acylation of 2Rs15d-H$_3$*K*H$_6$ with 20 to 40 equivalents of acylating reagent. Spectra are zoomed-in on the peaks corresponding to the three most abundant charge states (+9, +10 and +11). The peak with m/z of 1428.5 corresponds to the mono-acylated protein with charge state of +10. Neighbouring peaks with the same charge state, but belonging to starting material, di-acylated and tri-acylated product are labeled with 0, 2 and 3, respectively.

The strain promoted click procedure was optimized by testing different concentrations of added endo-BCN-PEG4-vc-PAB-MMAE: The click reaction between acylated 2Rs15d-H3KH6 and endo-BCN-PEG4-vc-PAB-MMAE (BroadPharm) was performed in 20 mM Tris pH 7.5 at room temperature (step 2, Figure S9). The payload endo-BCN-PEG4-vc-PAB-MMAE in DMSO (2.4 mM) was added to acylated 2Rs15d-H3KH6. To avoid precipitation of payload during these conditions, the co-solvents dimethyl formamide (DMF, 5%) and propylene glycol (PG, 20%) were found to be optimal additives to ensure complete dissolution of payload during the click reaction. Initially,

addition of various amounts of endo-BCN-PEG4-vc-PAB-MMAE was tested. Briefly, endo-BCN-PEG4-vc-PAB-MMAE (7-20 equivalents) was added to azidated 2Rs15d- H3KH6 (1.2 mg/mL) in 20 mM Tris (pH 7.5). Next, DMF (5% v/v) and PG (20% v/v) were added, and each reaction was adjusted to a final protein concentration of 0.7 mg/mL using 20 mM Tris (pH 7.5). The reaction mixture was degassed, and the reactions were shaken (500 rpm) on a thermomixer (37 °C) for 48 h. Next, excess endo-BCN-PEG4-vc-PAB-MMAE was removed using 7 KDa MWCO Zeba Spin pre-equilibrated with 20 mM Tris (pH 7.5), and each sample was analyzed by SDS PAGE (Figure S12).

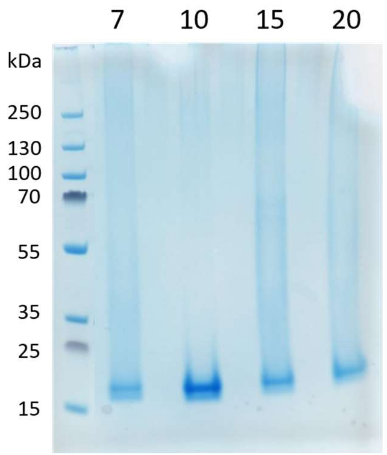

Figure S12: SDS-PAGE gel of the conjugation of endo-BCN-PEG4-vc-PAB-MMAE to azidated 2Rs15d-H_3*K*H_6. The numbers above the lanes indicated the equivalents of payload added to the protein.

Based on this initial screening 20 equivalents of endo-BCN-PEG_4-vc-PAB-MMAE were used in further and 2Rs15d-H_3*K*H_6 (0.5 mg) were used in the following up-scale reaction (see main article), which were purified by size-exclusion chromatography on a Superdex75 10/300 GL column (GE Healthcare) using PBS as eluent (Figure S13a). Fractions (0.2 mL each) were subjected to SDS-PAGE analysis followed by Western blotting using an anti-His antibody (Invitrogen) (Figure S13b). Fraction f21 containing the pure mono-functionalized conjugate, as confirmed by intact-protein MS analysis (Figure S14), was selected for cytotoxicity studies.

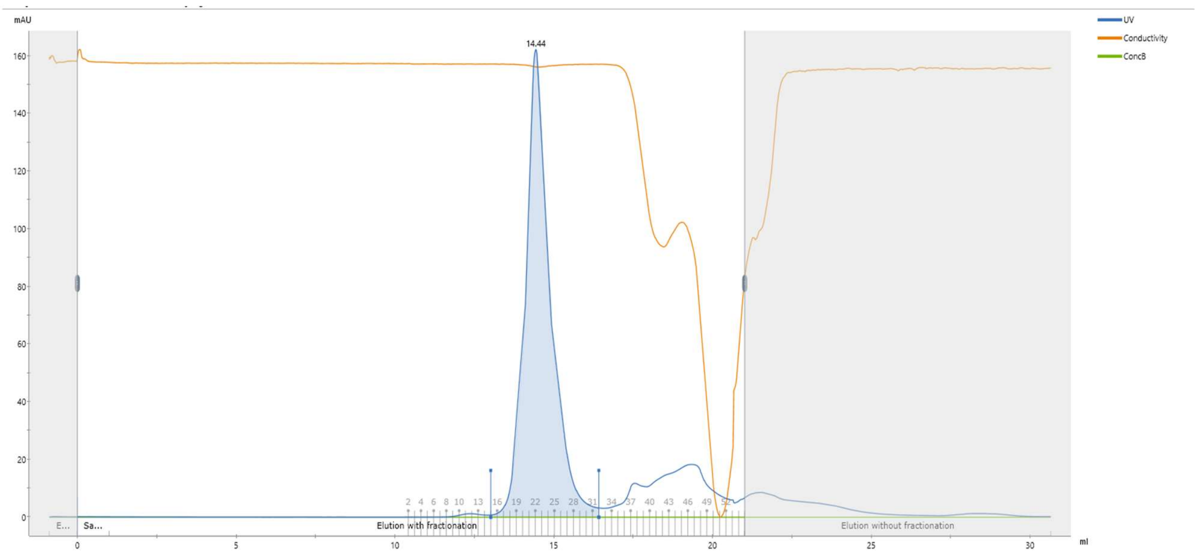

Peak Table - UV

Peak	Retention (ml)	Area (ml*mAU)	Area %	Ext coeff. (mg⁻¹ ml cm⁻¹)	Fraction(s)	Volume (ml)	Amount (mg)	Concentration (mg/ml)	Conductivity (mS/cm)
Peak A	14.438	147.0	100	1.800	15 - 31	3.402	0.408	0.120	23.00

Figure S13a: Purification of 2Rs15d-H_3*K*H_6-MMAE by size-exclusion chromatography.

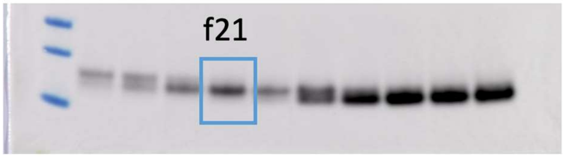

Figure S13b: Western blot analysis of fractions 18 to 27 with anti-His antibody. The volume of each fraction loaded on the gel was adjusted based on the respective UV intensity. (The lower intensity of the bands in fractions 18 to 23 compared to fractions 24 to 27 can be explained by the fact that the antibody binds less well to the His tag after conjugation of the payload.) In other words, it does not reflect the amount of protein in the respective fractions.

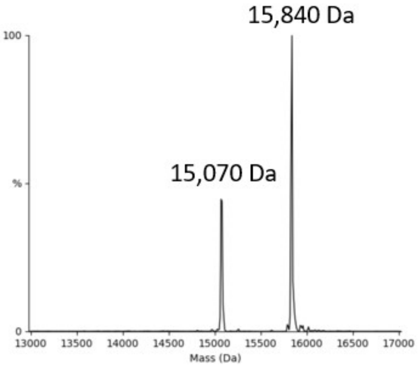

Figure S14: Deconvoluted ESI-TOF spectrum of 2Rs15d-H_3*K*H_6-MMAE. The calculated mass of the conjugate is 15822 Da.

Chymotrypsin digestion and LC-MS/MS analysis of acylated 2Rs15d-H_3KH_6

The crude reaction mixture containing 2Rs15d-H_3KH_6 and 4-methoxyphenyl 2-azidoacetate was digested with chymotrypsin (prepared in duplicate) and subjected to LC-MS/MS analysis using a 2cm C18 trap column (ThermoFisher 164946), connected in-line to a 15 cm C18 reverse-phase analytical column (Thermo EasySpray ES904) using 100% Buffer A (0.1% Formic acid in water) at 750bar, using the Thermo EasyLC 1200 HPLC system, and the column oven operating at 35°C (see main article). The numbers of PSMs covering the N-terminus and Lys residues in 2Rs15d-H_3KH_6, unmodified or acylated, are listed in table S2. The amino acid sequence of 2Rs15d-H_3KH_6 with Lys residues underlined is provided for reference.

Table S2. Numbers of PSMs covering the parts of 2Rs15-H_3KH_6 with unmodified and/or acylated N-terminus and Lys residues, respectively. For each peptide, two numbers are provided as the digest was prepared in duplicate.

Residue	#PSMs, unmodified	#PSMs, acylated
N-terminus, K21	38<br>21	0<br>0
K62, K66	3<br>3	1<br>0
K77, K78	12<br>14	3<br>4
K88	n.d. (1)	n.d. (0)
K121	0<br>0	5<br>5

[a] No PSMs were obtained for Lys residue 88 when the protein was digested in solution under non-reduced conditions. The number in between brackets reflects the number of PSMs obtained from a sample being digested in gel under reducing conditions instead.

```
1          11         21         31         41         51
MAEVQLQESG GGSVQAGGSL KLTCAASGYI FNSCGMGWYR QSPGRERELV SRISGDGDTW
61         71         81         91         100        101
HKESVKGRFT ISQDNVKKTL YLQMNSLKPE DTAVYFCAVC YNLETYWGQG TQVTVSSHHH
121
KHHHHHH
```

Cytotoxicity of 2Rs15d-$H_3\mathit{K}H_6$-MMAE in vitro cell viability assay

As described in the main article, the serially diluted 2Rs15d-$H_3\mathit{K}H_6$-MMAE and 2Rs15d-$H_3\mathit{K}H_6$ (unconjugated control) with a maximum final nanobody concentration of 10 nM (10% PBS v/v) were tested in an in vitro viability assay as compared to control conjugates with a maximum final conjugate concentration of 10 nM (10% PBS v/v). for either 4 days (see Fig. S15) or 7 days (Fig 3), at which point overall viability was evaluated by adding 15 µL CellTiter 96 AQueous One Solution Cell Proliferation Assay (MTS) (Promega). The 96-well plate was incubated for an additional 60 min, and the plate read at 490 nm (background subtraction at 630 nm), and viability calculated as the percentage of internally untreated control cells.

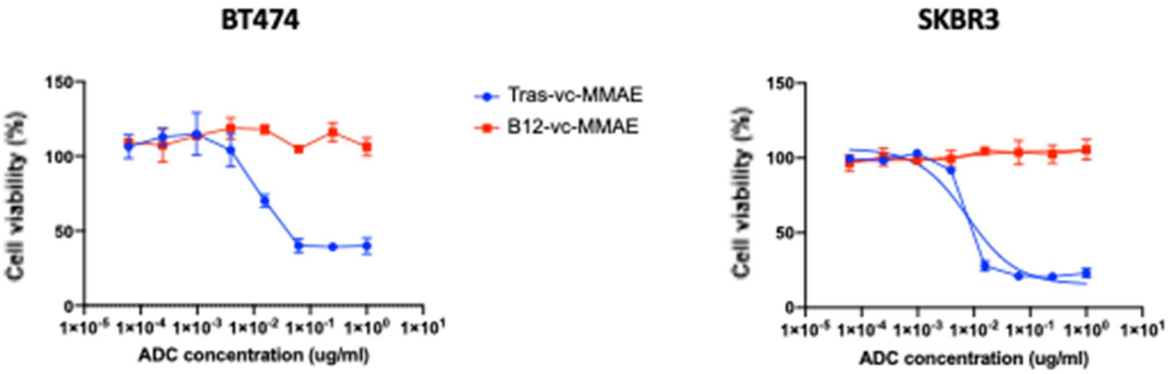

Figure S15: Anti-proliferative effect on HER2+ SKBR3 and BT474 cells after 4 days incubation with Tras-vc-MMAE and B12-vc-MMAE as control conjugates.

References

1 S. Massa, C. Xavier, J. De Vos, V. Caveliers, T. Lahoutte, S. Muyldermans and N. Devoogdt, *Bioconjug. Chem.*, 2014, **25**, 979–988.

2 M. D'Huyvetter, J. De Vos, C. Xavier, M. Pruszynski, Y. G. J. Sterckx, S. Massa, G. Raes, V. Caveliers, M. R. Zalutsky, T. Lahoutte and N. Devoogdt, *Clin. Cancer Res.*, 2017, **23**, 6616–6628.

3 C. Kofoed, S. Wu, K. K. Sørensen, T. Treiberg, J. Arnsdorf, S. P. Bjørn, T. L. Jense sn, B. G. Voldborg, M. B. Thygesen, K. J. Jensen and S. Schoffelen, *Chem. Eur. J.*, 2022, **28**, e202200147.

4 C. F. Nielsen, S. M. Van Putten, I. K. Lund, M. C. Melander, K. S. Nørregaard, H. J. Jürgensen, K. Reckzeh, K. R. Christensen, S. Z. Ingvarsen, H. Gårdsvoll, K. E. Jensen, P. Hamerlik, L. H. Engelholm and N. Behrendt, *Oncotarget*, 2017, **8**, 44605–44624.
